# The recurrence of odontogenic keratocysts in pediatric patients is associated with clinical findings of Gorlin-Goltz Syndrome

**DOI:** 10.4317/medoral.23185

**Published:** 2019-12-24

**Authors:** Leorik Pereira da Silva, Larissa Santos Amaral Rolim, Luiz Artur Barbosa da Silva, Leão Pereira Pinto, Lélia Batista de Souza

**Affiliations:** 1 DDS, MSc, PhD student, Oral Pathology, Postgraduate Program in Oral Pathology, Federal University of Rio Grande do Norte (UFRN), Natal, Rio Grande do Norte, Brazil; 2 DDS, MSc, PhD, Titular Professor, Postgraduate Program in Oral Pathology, Federal University of Rio Grande do Norte (UFRN), Natal, Rio Grande do Norte, Brazil

## Abstract

**Background:**

Odontogenic keratocyst (OKC) is an odontogenic developmental cyst that presents distinct clinical behavior. This lesion has been described as dental cysts with keratinization since the 1930s, however the term "OKC" was established in 1956. This study aims to determine the frequency and features of OKC in children aged 0 to 14 years in an oral pathology service in Brazil.

**Material and Methods:**

A retrospective study was performed to review cases of OKC in children diagnosed between 1986 and 2017. Clinical data were evaluated from medical records (gender, race, age, anatomical location, treatment, radiographic findings and follow-up).

**Results:**

Ninety-seven cases of OKC were diagnosed in a 31-year-period in all age groups and 10 were found in children (10.3%). Age ranged from 2 to 14 years (mean age=10.5±3.5), with 8 males and 2 females. The most frequent location was the anterior region of the mandible (n=4). Patients were predominantly asymptomatic. Moreover, in two children, clinical findings of Gorlin-Goltz Syndrome were observed. The most commonly used treatment was enucleation followed by curettage. In all cases of Gorlin-Goltz Syndrome were observed recurrences and occurrence of new keratocysts.

**Conclusions:**

Although uncommon in pediatric patients, OKC should be considered a differential diagnosis in cases of osteolytic lesions in gnathic bones. Thus, the periodic assessment of children by dentists and pediatricians is essential to get a correct diagnosis and early treatment to avoid greater mutilation of these patients.

** Key words:**Odontogenic cysts, children, odontogenic keratocyst, Gorlin-Goltz Syndrome.

## Introduction

Since the 1930s, researchers have noted keratinization in dental cysts. However, the use of the term "odontogenic keratocyst" was first suggested by Philipsen for all odontogenic cysts that presented keratinization in the epithelium (1). Odontogenic keratocyst (OKC) is a benign intraosseous cystic lesion of the gnathic bones which probably originates from the remnants of the dental lamina (2,3). In 2005, the classification of Tumors of the Head and Neck of the World Health Organization (WHO) classified this lesion as a neoplasm derived from the odontogenic epithelium, naming it as a Keratocyst Odontogenic Tumor (2-5). However, the recent WHO classification had gone back and reclassified this lesion as an odontogenic cyst.

This lesion has a slight preference for the male gender, occurring mainly between the second and fourth decades of life, usually affecting the posterior region of the mandible and ascending branch. At the imaging examination, it presents as a radiolucent lesion, unilocular or multilocular, well delimited, that may or may not be associated with an unerupted tooth. The diagnosis of OKC may only be obtained by biopsy and histopathological analysis. It is characterized by a stratified parakeratinized epithelium lining, typically thin and uniform thickness, free of inflammation, and presence of corrugated layer keratin. The basal layer is cuboidal or columnar with palisading (2-7). Treatment may be conservative or aggressive, but with lesions involving permanent non-erupted teeth in children and adolescents a less mutilating approach is recommended (3,7,8).

Most OKC appears as an isolated solitary lesion. However, this cyst may be associated with Nevoid Basal Cell Carcinoma Syndrome (NBCCS), or Gorlin-Goltz Syndrome, which is a rare autosomal dominant syndrome characterized primarily by multiple/recurrent OKC (present in at least 75% of patients), basal cell carcinomas and other clinical findings which usually appear during puberty or even during the first decade of life (5,9,10).

The incidence of OKC is uncommon in patients from zero to 14 years, and few studies in literature have reported cases in non-syndromic children (11-16). Based on that, the aim of this retrospective study was to review cases of OKC in children that attended an oral diagnostic center in Brazilian Northeast between 1986 and 2017 and to evaluate the presentation, radiological findings, and outcomes.

## Material and Methods

The files and histological material retrieved from the Oral Pathology Service archive of UFRN were reviewed. Data from patients with OKC were obtained over a 31-year-period ][1986-2017]. Cases of OKC were selected in children aged zero to 14 years. This retrospective study was approved by the Local Research Ethics Committee (protocol No. 1.768.092).

A total of 1,060 cases of patients aged ≤ 14 years were analyzed. Data such as gender, race, age, anatomical location, treatment, radiographic findings, recurrence and histopathological diagnosis were compiled for all cases from the clinical data sent together with the biopsy records.

## Results

There were 97 cases of OKC during the period studied; of these, 10 cases (10.3%) occurred in children less than 14 years (mean age=10.5±3.5) and were reassessed for the present study. Based on the population of children diagnosed with oral and maxillofacial lesions (n=1060), the prevalence of OKC was 0.95% and the clinical findings of Gorlin-Goltz Syndrome were approximately 0.2%. The cases were more common in males with the male:female ratio of 4:1. The most frequent location was the anterior region of the mandible with mandible:maxilla ratio of 2.3:1 (Table 1).

**Table 1:**
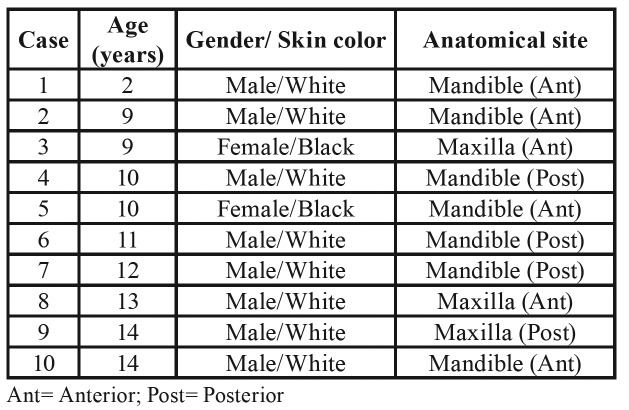
Characteristics of odontogenic keratocysts in pediatric patients diagnosed in 31 years in a referral service in oral and maxillofacial diagnosis.

The lesions were radiographically well delimited and mainly unilocular (n=8). The lesions were asymptomatic in 7 cases and the size of cysts varied between 2 and 9 cm. The main diagnostic hypothesis was dentigerous cyst and most of the lesions were associated with impacted teeth, primarily canine teeth (n=4). Most patients were treated with enucleation and curettage (n=5) (Table 2).

In two patients (Cases: #5 and #10), besides the diagnosis of OKC, it was observed clinical features suggestive of Gorlin-Goltz Syndrome (also called nevoid basal cell carcinoma syndrome), such as hypertelorism, mandibular prognathism, mental retardation and strabismus. Due to lack of financial resources in the public health service, it was not possible to confirm mutations on patched gene (PTCH), which is a tumor suppressor gene, located on chromosome 9q22.3-q31. It is important to emphasize that post-treatment OKC recurrences reported in the present study were found in the two patients with signs of Gorlin-Goltz Syndrome. The patients presented a second OKC 4-6 years after first diagnosis in the posterior region of the mandible associated with third molar.

**Table 2:**
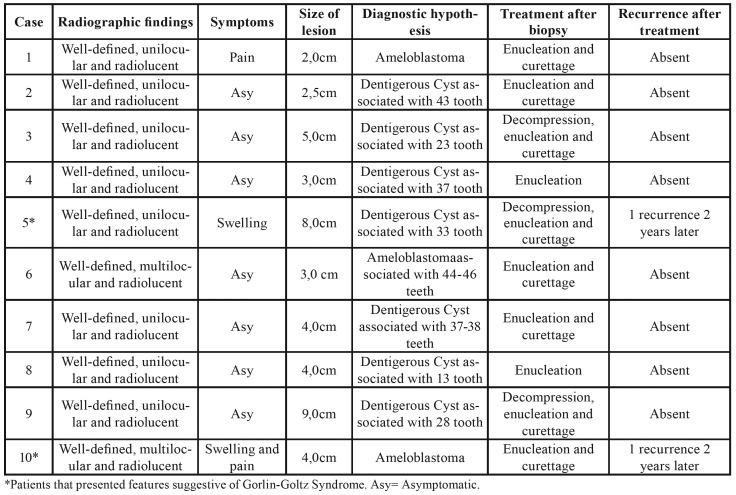
Clinical and radiographic findings of odontogenic keratocysts in pediatric patients diagnosed in 31 years in a referral service in oral and maxillofacial diagnosis.

## Discussion

Of all odontogenic cysts, OKC represents about 6 to 25.7% and appears to be more frequent in males with a peak incidence of 20-30 years of age. This lesion deserves differential considerations due to its clinical presentation, since it presents invasive-infiltrative growth, expanding in great proportions, causing considerable bone destruction usually before any symptoms (5,8,17-19).

Patients with Gorlin-Goltz Syndrome present several clinical presentations that may aid in the diagnosis. Some sets of diagnostic criteria were proposed, but the criteria of Kimonis *et al*. (20) seems to be the most pertinent, being composed of six major criteria and six minor ones. Patients with more than two basal cell carcinomas, presenting OKC in the gnathic bones histologically diagnosed and bilamellar calcification of the falx cerebri are some of the major criteria, while congenital malformations, such as cleft lip or palate, frontal bossing, “coarse face”, moderate or severe hypertelorism, are some of the minor criteria. In two patients of our study presented features suggestive of Gorlin-Goltz Syndrome, such as hypertelorism, mandibular prognathism and histological diagnosis of OKC. These clinical characteristics found in our patients, in addition to the recurrences they presented, may be indicative they have Gorlin-Goltz Syndrome.

In the present study, ten children diagnosed with OKC were found to be up to 14 years of age, representing 10.3% of all OKC. In addition, less than 1% of all maxillofacial lesions diagnosed in these patients during the period studied, with two cases associated with clinical features of Gorlin-Goltz Syndrome. This fact corroborates with the literature, which points out this lesion as extremely rare in pediatric patients (8,11,12,16).

The presentation of multiple OKC or associated with other lesions is very rare in non-syndromic patients. However, Arcuri *et al*. (16) reported an unusual case of OKC which presented simultaneously with a pleomorphic adenoma on the palate of an 11-year-old male patient. In addition, Ozkan *et al*. (13) described a case of a 12-year-old non-syndromic female patient who presented multiple OKC in multiple regions of the mandible and maxilla. This author described that treatment consisted of enucleation of the lesion of the anterior region combined with marsupialization of the extensive lesions in order to avoid bone fractures or impairment of important anatomical structures.

In gnathic bones OKC has a diverse incidence, with posterior region of the mandible being the most affected site (2,13,15,16). However, in our study anterior region of the mandible was the most common anatomic site followed by posterior region of the mandible and anterior region of maxilla.

The most common symptomatology reported in OKC consists of swelling and pain, although in most cases it is asymptomatic in early stages. Radiographically, this lesion presents unilateral or multilocular radiolucency, occasionally expansive (7,15). In our study, lesions were asymptomatic and presented unilocular radiolucent lesions similar to those reported in the literature (2,14-16).

In general, the recurrence rate of OKC is variable, from 5 to 70%. Several theories have been raised regarding the cause of lesion recurrence, which include the incomplete removal of the original cystic lining, growth of daughter cells present in the cystic capsule or odontogenic remnants that were left after surgery (2,3). In addition, the mutation in the patched gene present in the Gorlin-Goltz Syndrome predisposes the recurrence and multiple cysts and tumors in the affected patients.

In the study conducted by Zecha *et al*. (7), it was assessed the recurrence rate in 68 patients with non-syndromic OKC in a 34-year-period. The patients in this study were submitted to two conservative surgical treatments: enucleation and marsupialization. These authors observed that out of 54 patients who underwent enucleation, 12 presented recurrence, corresponding to 20.7%, while four out of ten patients who underwent marsupialization, had recurrences, corresponding to 40%, showing a higher rate of relapse of these two treatments. Cunha *et al*. (3) evaluated the aspects associated with recurrence of non-syndromic OKC, and out of 24 patients previously treated with enucleation followed by peripheral osteotomy, eight patients developed recurrent lesions in a follow-up period of two years, representing a 33% rate of relapse.

The recurrence of these lesions in children has not been well documented; however, Hyun *et al*. (2) reported a case of recurrent OKC in a 7-year-old male patient who presented a lesion in the right mandibular body. The initial treatment recommended by these authors was marsupialization, followed by curettage, and only after relapse of the lesion a complete enucleation was performed. In general, recurrences are not commonly described in pediatric non-syndromic patients.

Therefore, it must be emphasized that the stage of development and eruption of the tooth need to be considered for the therapeutic planning of pediatric patients (20). Infants with permanent, non-erupted teeth need a more conservative surgical treatment whenever it is possible (2). In the present study, none of the non-syndromic pediatric patients (n=8) presented recurrence after conservative treatment in long follow-up. A follow-up for a long period is necessary to prevent recurrent lesions from being diagnosed late and leading to greater mutilation.

We show in the current study specific characteristics of OKC in children younger than 14 years, since in a study previously published by our research group (21), we understood that the presentation and follow-up of these cysts needed to be detailed. In general, there was a greater occurrence of OKC in children in anterior region of the mandible and the recurrence rate of the lesion after conservative treatment was related to patients with the Gorlin-Goltz Syndrome.

## Conclusions

The occurrence of OKC in pediatric patients with or without Gorlin-Goltz Syndrome is uncommon, although it should be considered as a differential diagnosis in cases of osteolytic lesions in gnathic bones. The periodic assessment of children by dentists and pediatricians is essential so the diagnosis of this lesion can be done quickly, and an adequate and less aggressive treatment is feasible. The absence of a tooth in the clinical examination should be investigated by radiographic examination, since OKC is infiltrative, asymptomatic and associated with an impacted tooth in most cases. The presence of clinical findings of Gorlin-Goltz Syndrome should be observed in children, since cystic lesions present a higher risk of recurrence.
